# Problems maintaining collaborative approaches with excluded populations in a randomised control trial: lessons learned implementing Housing First in France

**DOI:** 10.1186/s12961-018-0305-1

**Published:** 2018-04-19

**Authors:** Pauline Rhenter, Aurélie Tinland, Julien Grard, Christian Laval, Jean Mantovani, Delphine Moreau, Benjamin Vidaud, Tim Greacen, Pascal Auquier, Vincent Girard

**Affiliations:** 1 Aix-Marseille University, Public Health Research Unit EA 3279, 9 rue Dragon, 13006 Marseille, France; 2 Community Mental Health Outreach Team, MARS (Movement and Action for Social Recovery), Public Hospital of Marseille (AP-HM), Marseille, France; 3 Observatory Regional of Health, Midi Pyrénées, Toulouse, France; 4 Research Laboratory, Maison Blanche Hospital, Paris, France; 5 Regional Agency of Health (Agence Régionale de Santé PACA), Marseille, France

**Keywords:** Coalition, Civil disobedience, Collaborative research, Evidence-based policy, Housing First, Knowledge translation, Social movement, Window of opportunity

## Abstract

**Background:**

In 2006, a local collective combating homelessness set up an ‘experimental squat’ in an abandoned building in Marseille, France’s second largest city. They envisioned the squat as an alternative to conventional health and social services for individuals experiencing long-term homelessness and severe psychiatric disorders. Building on what they learned from the squat, some then joined a larger coalition that succeeded in convincing national government decision-makers to develop a scientific, intervention-based programme based on the Housing First model. This article analyses the political process through which social movement activism gave way to support for a state-funded programme for homeless people with mental disorders.

**Methods:**

A qualitative study of this political process was conducted between 2006 and 2014, using a hybrid theoretical perspective that combines attention to both top-down and bottom-up actions with a modified Advocacy Coalition Framework. In addition to document analysis of published and grey literature linked to the policy process, researchers drew on participant observation and observant participation of the political process. Data analysis consisted primarily of a thematic analysis of field-notes and semi-structured interviews with 65 relevant actors.

**Results:**

A coalition of local activists, state officials and national service providers transformed knowledge about a local innovation (an experimental therapeutic squat) into the rationale for a national, scientifically based project consisting of a randomised controlled trial of four state-supported Housing First sites, costing several million euros. The coalition’s strategy was two-pronged, namely to defend a social cause (the right to housing) and to promote a scientifically validated means of realising positive outcomes (housing tenure) and cost-effectiveness (reduced hospitalisation costs).

**Conclusion:**

Activists’ self-agency, especially that of making themselves audible to public authorities, was enhanced by the coalition’s ability to seize ‘windows of opportunities’ to their advantage. However, in contrast to the United States and Canadian Housing First contexts, which are driven by implementation science and related approaches, it was grassroots activists who promoted a scientific-technical approach among government officials unfamiliar with evidence-based practices in France. The windows of opportunity nevertheless failed to attract participation of those most in need of housing, raising the question of whether and how marginalised and/or subordinate groups can be integrated into collaborative research when a social movement-driven innovation turns into a scientific approach.

**Trial registration:**

The current clinical trial number is NCT01570712. Registered July 17, 2011. First patient enrolled August 18, 2011.

## Background

In 1992, a new intervention model was developed in New York City to respond to the problem of homelessness among people living with severe psychiatric disorders [1]. Within the decade, data from Housing First randomised controlled trial (RCT) supported its effectiveness and efficiency [2]. The model was disseminated throughout the United States, mainly through cutting-edge RCT designs [1], transforming Housing First into an evidence-based policy [3]. In 2009, Canada implemented a large, multi-site Housing First RCT design at a cost of over CAN$ 125 million with a sample size of 2300 study subjects [4]. In 2011, France, a country then unaccustomed to evidence-based policy, became the first European country to implement a large, multisite Housing First RCT inspired by the Canadian model [5]. A major impetus was the knowledge gained from a local therapeutic experiment in an illegally occupied building, or squat, for people with long-term homelessness and severe mental disorders.

Developing innovative practices at the local level raises the question of whether these can be disseminated to other contexts [6], as well as whether local initiatives can be translated into national-level policies. However, the starting point for Housing First was not the same in France as in North America. In the United States, Housing First originated after national legislation had already funded proto-typical programmes and evidence of their effectiveness had been partly established [7]. In Canada, the Housing First initiative, At Home/*Chez Soi*, has been attributed to the ‘policy entrepreneurship’ of a powerful Senator able to take advantage of favourable contextual factors [4]. This paper presents a sociopolitical analysis of a contrasting case. In France, local activists were able to push for evidence-based approaches that elsewhere – but not yet in France – drove government-funded policy on mental health and housing. This paper analyses the process through which a broad coalition achieved Ministry-level support for the largest randomised experiment of its kind in Europe, financed by a several million euros state budget [8]. The case study also raises the question of who participates and who is left out in this broad process of policy formation.

### Theoretical framework

This paper’s theoretical framework draws on constructivist critiques [9] of policy transfer studies that counter the rationalist-linear and technicist notion of policy as ‘products’ and ‘events’ flowing top-down from decision-makers or centre-periphery from experts. Rather than defining policy as ready-made ideas to be implemented, the constructivist approach views it as the result of often chaotic processes of reciprocal knowledge exchange, actions and adjustments between actors [10]. Our analysis of the political process through which the French Housing First programme came about adopts this dynamic approach within a hybrid theoretical framework for capturing agency and domains of action. First, it takes into consideration both top-down expertise [11] and the value-based, bottom-up actions of community groups [12]. Second, it draws on a modified Advocacy Coalition Framework (ACF) that incorporates the critiques of French political scientists [13]. ACF considers policy to be a cumulative sequence of interactions between multiple actors from different contexts and levels. Using specific French examples, Bergeron et al. [13] challenge the functionalist ACF conception of a relatively stable policy subsystem, which disallows the possibility of a paradigmatic shift. Thus, by adopting a hybrid framework, our analysis remains attentive to the multiple sites where policy is experimented, to the heterogeneity of actors in relation to power, and new heterogeneous domains of public action and policy networks [14].

## Methods

The study methodology involved a grounded theory constructivist approach [15], in which hypotheses are constantly reformulated throughout the recursive process of data collection and analysis. By equally privileging the points of view and situatedness of all actors, this methodology allows us to identify how all stakeholders appropriate or resist new policy and how their actions flow from particular interests and power relationships at hand. Specifically, the study distinguished the categories of actors involved and the heterogeneous worlds they came from (experts/community, central organisations/local authorities and activists, etc.) and the transactions that gave shape at given moments to alliances between actors in support of their involvement in collaborative processes. We also attempted to characterise the aspects through which actor beliefs and interests crystallised in the implementation of Housing First/*Un Chez Soi d’Abord*, or ‘first a place of one’s own’ (henceforth *Un Chez Soi d’Abord*) and the formation process of actor coalitions. The study of the political process was made by three sociologist researchers and one political scientist researcher.

Three data sources were created, as follows:

1. For document analysis, documents directly and indirectly linked to the implementation of the experiment were collected. These included steering committee and research meeting minutes, guidelines, responses to calls for tender, internal regulations, national and local media releases, reports, and scientific and media articles. The corpus of documents was comprehensive; that is, no selection was made.
2. Field notes were kept of participant observation and observant participation. Participant observation is the classical ethnographic method of immersion, systematic observation and note-taking by an outsider of actions in the world being studied. From 2007 to 2011, the political scientist conducted participant observation of the programme’s governance mechanisms, namely local and national steering committees. Observant participation is carried out by a ‘natural’ insider or participant who, by doing so, becomes a source of data [16]. One researcher had been a key player in public actions from the beginnings of the experimental squat through the functioning national RCT in 2014 [17].
3. Semi-directive interviews were conducted with 65 individuals involved at different levels and stages of the *Un Chez Soi d’Abord* project. Interviewees were identified by five key players initially interviewed, and by the political scientist on the basis of her observations of meetings at the national level and in Marseille. They included hospital directors, directors of housing and social integration non-governmental organisation (NGOs), local government representatives in the health and social fields, social housing providers, hospital psychiatrists, spokespersons and chairpersons of housing and mental health user groups, researchers involved in programme assessment, members of local steering committees in each of the four cities involved, members of teams partnering with the programme to recruit people into the study, and activists from the experimental squat in Marseille.

Document analysis, participant observation, recording of field-notes and interviews were conducted by three sociologists and a political scientist. Data analyses were conducted by the four researchers between 2012 and 2015, and consisted of thematic analyses of the corpus of the documents, field notes and interviews, beginning with the document analysis. Results from document analysis contributed to questions asked in subsequent semi-structured interviews. All materials were read numerous times for familiarity, to identify themes, events, dates and actors, and to cross-reference themes between types of data (e.g. themes in interviews with themes in meeting summaries). Themes were then coded, combined and contrasted to develop networks of associations. These were checked back with the other researchers, who met annually to discuss ongoing data collection and compare their respective data.

### Background of the French *Chez Soi D’Abord* RCT

The Marseille experimental squat was established during a period of nationwide social movement activism against homelessness in France. In 2006, the NGO *Médecins du Monde* [Doctors of the World] provided hundreds of tents to homeless people all over France. The following year, activists calling themselves ‘Children of Don Quixote’ installed new tents [18], amplifying the media message and achieving the movement’s first goal – to make homelessness visible to the public. A year later, this mobilisation, which by then included most groups involved in the field of homelessness, won a second major victory when it persuaded the government, then facing a presidential election, to pass legislation making housing a universal right for all French citizens [19].

Also in 2006, the Collective in Marseille, France’s second largest city (pop. 852,516) and one of its poorest [20], employed civil disobedience to illegally occupy a building and open it to people experiencing long-term homelessness, severe psychiatric disorders and/or addictions [21]. This act, carried after consultation with a housing rights attorney, culminated months of preparation by the collective’s members, including mental health workers, other professionals, activists and homeless individuals. Within weeks, the building was transformed into an experimental therapeutic squat, with a living and treatment space serviced by an outreach team, to provide an alternative to conventional homeless shelters and psychiatric hospitalisation. The outreach team was connected to a university psychiatric teaching facility and had considerable experience in street work aimed at building trust among homeless people with psychiatric and addiction conditions [22]. Eighteen months after the illegal occupation, the city of Marseille legalised the squat [21].

In October 2008, the experimental squat’s outreach team presented its first paper in a scientific venue, a national congress on health inequalities, reporting the positive effects of access to housing on the reduction of health inequalities. An advisor from the Ministry of Health in attendance then convinced its Health Minister to visit the squat, with the visit leading to two decisions from the Health Minister several weeks later, namely to allocate substantial financial resources to the psychiatric street outreach team, and to request its head psychiatrist to conduct a national report on the health of homeless people. The Report was submitted in 2010 and highlighted, among other problems*,* the fact that most French services to end homelessness use a step-by-step, ‘treatment first’ approach to access to housing, for which they also require abstinence from substance use. Paradoxically, these criteria result in the exclusion of those individuals with the most problems [23].

The Health and Housing Ministries then decided to accept one of the Report’s major recommendations by agreeing to establish a Housing First-type programme for long-term homeless people with severe psychiatric disorders [8]. The subsequent national multi-site (Marseille, Lille, Paris, Toulouse) RCT, based on the United States and French RCTs, came into being in August 2011 [24], just after the 2012 French presidential elections. In what follows, we provide an in-depth sociopolitical analysis of the implementation of the French *Un Chez Soi d’Abord* RCT.

### Initial data analyses and new questions

The analysis of the process through which the RCT came about is reconstructed from data gathered over several years after the decision to establish the programme.

From December 2011 to February 2012, researchers conducted five exploratory interviews with national-level actors involved in the elaboration of the French *Un Chez Soi d’Abord* since 2009. These included the director of a Ministry, three experts on street outreach and the social worker-coordinator of a programme for direct access to housing, who were asked to tell the story of how the *Un Chez Soi d’Abord* programme was started. They were then asked their reasons for becoming involved with this RCT, how different aspects of the projects were conceptualised, about obstacles and factors facilitating implementation at local and national levels, and about key actors involved in the implementation.

From 2012 to 2014, researchers focused on understanding the context in which the Housing First/*Un Chez Soi d’Abord* programme had first come into being. To do so, the political scientist interviewed 40 key stakeholders identified in the exploratory phase, including NGO and health establishment directors involved with the four programme sites, *Un Chez Soi d’Abord* coordination teams, partners from the fields of psychiatry, addictology, housing and social integration, programme monitors from other state services, funding agencies, social housing property owners, and representatives of health and housing users. These were supplemented by the analysis of documents gathered. The analyses revealed that the origins of *Un Chez Soi d’Abord* were made possible by the formation of a public action network organised around “*new domains of public action*” [14], so called because they brought together intellectual and practical resources as well as categories of knowledge and action from different sectors (housing, health, welfare, research) and mobilised a plurality of public, private, national and local actors. The network was further explored through the document analysis of the governance-related tools and devices it produced, namely calls for tender, research protocols, internal regulations, conventions, media releases, press releases, budgetary instruments, and minutes from local and national steering committee meetings. In addition, the political scientist collected pre-programme implementation planning documents from each of the four *Un Chez Soi d’Abord* RCT sites. She took notes at local and governmental meetings from which she developed 46 summaries, and of real-time sociopolitical observation of eight inter-ministerial meetings.

In 2014, after reviewing data gathered so far, the research team decided to deepen their analysis by turning their attention to actors who were less visible than those officially responsible for the RCT in order to retrace how the experimental squat in Marseille had come into being. This final series of interviews was conducted with members of the Marseille Collective and actors working for centralised state services (*n* = 10). The goal was to understand the roles of these ‘minor actors’ in transferring knowledge that contributed to legitimising the RCT programme (as *Un Chez Soi d’Abord* was supported by the national government, its final characteristics drew only partially from the Marseille experimental squat). Actors interviewed were the inter-ministerial delegate responsible for the implementation of the RCT programme, the national coordinator of the intervention wing of the programme, the university professor and director of the research centre responsible for evaluating the intervention, the Health Minister who had first involved the government in the RCT, three national administration agents who had participated in the negotiations between the ministers involved with the programme and drawn up the terms of reference for the intervention wing, and three activists who had participated in the Health-Housing Collective and the experimental squat between 2007 and 2009.

## Results

The genesis of the French *Un Chez Soi d’Abord* programme evolved in two separate phases, each of which engaged a different set of actors. The programme began when squatter activists armed with the user-based knowledge they had accumulated and claims to the right to housing adopted the political strategy of documenting and evaluating the therapeutic value of having access to a home. Their attempts ended quite improbably when, as a result of negotiations between government services *Un Chez Soi d’Abord* found a place on the political agenda as an economically feasible and promising RCT, enhanced by the use of controlled experimentation to evaluate public action [25].

### Civil disobedience as a strategy for public legitimisation of a social problem

As noted above, the experimental squat was founded in an act of civil disobedience, namely the illegal occupation of an abandoned building, to which members of an activist collective then provided services. In 2007–2008, the squat took in some 40 individuals to whom a local street outreach team had been providing support and who met the admission criteria of having a severe mental disorder, having had ineffective and/or unsatisfactory contact with the health system, and living on the street. The squat became a community living space, with cultural and artistic activities. The Collective, however, tended to play down its existence in the media and with neighbours.

The 2008 Activity Report described the first 40 individuals quantitatively:

“[…] *among 40 persons who spent at least one night at the squat within the past year, 22 (55%) came directly from the streets, 10 (25%) were referred by* [organisational] *partners, and 5 (12.5%) came from psychiatric hospitals. In terms of prevalence of psychiatric disorders, 16 (40%) had schizophrenia (of whom 65% had a comorbid addiction), 33% had a mood disorder (91% with comorbid addiction), 6 (15%) had three or more psychiatric diagnoses, 29 (72.5%) had previously consulted psychiatrists, 30 (75%) had undergone psychotherapy, 28 (70%) had received psychiatric medication, 38 (95%) health education and 57% had consulted a specialty service within a hospital… Among these, over the preceding year, 20% left the streets permanently and became engaged in a process of recovery and acquiring social skills.*”

Within months of the experimental squat’s founding, members of the Collective connected up with heads of the city’s public health services, with the aim of negotiating the legalisation of the squat. A contextual condition is important to understanding how legalisation was made possible. Although in France, health services are financed by the central government, the origins of the Marseille public health service are particular [26]. The municipal public health service was headed by two individuals who saw its work as the continuation of the 1990s harm reduction movement, but applied to the poverty and mental health fields of the present. Their understanding of the problems and proposed alternatives was similar to that of the Collective. In particular, the presence of a former homeless person in the Collective, the value attributed to notions such as self-help and empowerment, and the use of civil disobedience as a means of claiming fundamental human rights were perceived as qualities associated with the ideological heritage of the harm reduction model. Thanks to negotiations led by the two Marseille municipal public health administrators, the Collective and a university psychiatry professor, the Mayor legalised the squat.

One of the administrators later explained his support for the legalisation in these terms:

“*The history of AIDS and addiction is such that we were intensely engaged with the question of harm reduction, and that means several things: the fact of having had to build something with others, of reaching out to the most marginalised and working on social cohesion and marginality through health and social approaches, and also having to work in a very experimental mode rather than with already established models. This background really helped us understand how to approach the mental health field, and it soon became obvious that the two approaches had many issues – and people, too – in common.*”

On a practical level, the municipal public health service went on to play the role of mediator between the Collective and the municipal housing office, but also with the national government health services.

That the squat was an activist experience rooted in civil disobedience posed a problem in its evaluation. From the beginning, the squat’s supporters, namely clinicians, social workers, homeless people and junior researchers, used evaluation as a strategic means of producing evidence to support their demands. Although their principle objective was to bring about the recognition of the right to housing and treatment of homeless persons as a public problem requiring political solutions, they rallied around the immediate economic cost-benefit of the experimental squat, arguing that it avoided hospitalisation in some cases and reduced the number hospital days in others.

However, the squat supporters shared other, more operational goals. The first was to argue convincingly that the habitat they proposed facilitated access to effective care for people who were homeless and living with severe psychiatric disorders. A second, broader objective was to propose an experimental alternative to the then public and state-funded services and facilities for homelessness and for mental illness throughout France. The activists criticised what they saw as institutional paternalism and infantilisation, seeking instead the recognition of the competencies and strengths of individuals who had experienced homelessness, mental illness and their consequences – strengths which they could use and build on, and which until then had been ignored by the existing aid system [23]. Hence, the experimental squat was laying claim to the status of a social laboratory where people could search for solutions by and for themselves [21]. In interviews, it becomes clear that, without the squat as a forerunner, the *Un Chez Soi d’Abord* programme would not have developed in France. Yet, from the viewpoint of the proponents of this first phase, it would be inaccurate to posit this sequence of events as initiating an evidence-based policy. Rather, it involved the political recognition of a public problem, one that sought to integrate and assert a plurality of competencies, especially of the people who were most directly affected. As one of the squat’s first residents recalled: “*In June of 2004, I met the homeless mission of Doctors of the World and the psychiatrist told me what he had just done at Yale, where he was working with a mixed team – health professionals and psychiatric users who had lived on the streets – and I was one of the first. He told me: ‘you have real competencies’.*”

In the end, the *Un Chez Soi d’Abord* programme maintained two characteristic traits of the experimental squat that preceded it. The first was the strategy of approaching public problems through the theme of health, and particularly mental health. The second concerned the need to take the evaluation of its activities seriously, namely, of the ‘civil disobedience’ alternative in the French context, where the psychiatric field has been historically reluctant to accept evaluation [27].

The transition between these two moments – local experimentation and its national uptake – poses two corollary questions regarding the relationship between scientific expertise and politics, and regarding the part played by the users themselves in the intervention process, whether in the context of the social experiment or of the scientific experimental model that followed. Yet, as we will now see, while bottom-up knowledge produced from this local experiment paved the way for broader experimentation, in the long run, it was the top-down work of actors from the central government administrations which led to the implementation of a nationwide RCT.

### Furnishing evidence: a political strategy of the State

Even if social movements in health sometimes achieve their goals by judiciously exploiting international scientific discourse [28], scientific arguments alone cannot make social problems visible [29], let alone influence public policy concerning the social problems at hand [3]; ‘extra-scientific’ factors need to be taken into account [30]. Immediate and broader structural contexts are required to place scientific arguments on a political agenda.

In the present case, during the almost 2-year initial phase, supporters of *Un Chez Soi d’Abord* received little support from central government state agents. Few actors from the relevant ministries became involved. Nevertheless, those who did succeeded in supporting the experimental project by using translation strategies aimed at ‘convincing the unconvinced’, particularly those who controlled the financing of this experimental intervention and its evaluation. By translation, we refer to the ways in which an idea, object, action or interaction is contested and reformulated as it is detached from one sphere of action and reformulated and adapted to another.^1^

Translation was by no means a simple process, as can be seen in the lively debates that ensued at national government level. Before funding came through, the commitments made by the then Minister of Health had provoked a series of exchanges at the level of central government. The construction of the *Un Chez Soi d’Abord* experiment and its funding resulted from intense negotiations within the state apparatus, during and after the 2010 Report on homelessness and health, raising questions such as under which central government branch the programme fell and who could legitimately fund it. As one ministry official noted.

“*Madame Y, in the Sécurité Sociale* [Public Health Insurance] *branch, doesn’t understand that they don’t only fund healthcare. We don’t have the same cultural values as the people in the Health Department. For them,* [the RCT] *had to do with the Psychiatry and Poverty Outreach Teams. We said ‘no: it’s socio-medical, it’s pluridisciplinary, and you have to take the social component into account’. For the different administrations, the issue was: ‘Is this just an extension of the Psychiatry and Poverty Outreach Teams?’ With the Health and the Sécurité Sociale departments, it was: ‘Is this worth putting money into?’.*”

Within each of these administrations, actors who favoured the project took ownership of the research dimension and exploited it to gain influence and legitimacy within their own departments. They called on the *Institut de Recherche en Santé Publique* (the IRESP, or Public Health Research Institute), an intergovernmental, cross-institute research ‘meta-body’, to strengthen the scientific legitimacy of the project, and used this organisation to rally their more reticent colleagues to the idea of funding an evaluation. A scientific argument could compensate for prioritising a specific group, namely homeless persons with psychiatric disorders. Within the falling logic of the rationalisation of budgetary decisions and of the French universalist culture, numerous civil servants and central government officials gave low priority, thus benefitting a narrow target population. As detractors of the *Un Chez Soi d’Abord* programme, they used two types of arguments to defend their position. First, they stated that the programme did not fall within the scope of their jurisdiction (“*It’s is social welfare, whereas we work on healthcare and public health*”; “*It’s healthcare, but our job has to do with homelessness*”). The coordination of partners from different fields was even more complex when those concerned with welfare did not even consider the target population to be a priority: “*We have poor people who aren’t crazy!*”

Thus, several factors affected the position of state officials vis-à-vis the project, such as their department’s general culture, sub-cultures within each department, and their own professional trajectories. One civil servant, for example, suggested that the scientific aspect of *Un Chez Soi d’Abord* was made more strategic because she could relate it to the ‘field epidemiology’ she had conducted during her career: “*I was game from the very start because it was a unique approach and off the beaten path, and we had already tried so many things that didn’t work! But, then, to get the Department involved, you had to use a research strategy! … That was a time when the administrators were very resistant, which gave us the idea of funding research, especially the qualitative aspect … We knew it was going to be tough* [to support the programme]*, I mean, just think: crazy homeless alcoholics, and we were giving them money!*”

Another official, who had worked in the national programmes on AIDS, prisons, mental health and emergency care recalled enthusiasm for experimentality:

“*What I thought was unique and exciting about it was the notion of recovery, of harm reduction. We were failing with these groups. What I like is the innovative aspect. It’s interesting to accompany change, to see how the Americans had done it …* […] *Madame X, over in the social welfare administration, her position was, ‘let’s do it, we’ll find the money, this is activism’.*”

In the social welfare administration, as had happened with Marseille public health officials, what limited support existed was motivated by the ideological kinship between *Un Chez Soi d’Abord* and harm reduction: “*I’m originally a toxicologist. In the 1990s, I tackled HIV* […]. *Anyway, you have to take risks. ‘You take a good psychiatric patient, you put him in normal housing and if you do the right follow-up, he’ll lead a normal life?’ People said, ‘You’re nuts! You won’t get any results and it’s going to cost a fortune’.*”

These ‘rationally argued alliances’ developed more or less formally among actors from various backgrounds. The determining coalescing factor was a shared common belief that what was at stake in *Un Chez Soi d’Abord* were inequalities in health and access to healthcare. The then Minister of Health, who shared this conviction, summed up her political orientation in these terms:

“*The issue of social inequalities in health can only put a strain on a Health Minister. The health system creates enormous neglect, which can start to look like horrific mass relegation … I think the tremendous progress we’ve made in life expectancy and good health has to do, above all, with things like good nutrition, appropriate housing, education, and so forth. In short, the social perspective on health allows for huge advances, which are incredible reservoirs for improving health.*”

The support is more notable given the particular cultures of the different ministerial departments. The officials most receptive to the development of the *Un Chez Soi d’Abord* type RCT (who also happened to belong to those departments with the least funding discretion) tended to prefer qualitative evaluation. Yet, they valued the experimental and evidence-driven aspect of *Un Chez Soi d’Abord* and thought they could justify it as an exception to the institutional culture of the central administration missions. Similarly, the supporters of the Marseille squat proposed an RCT methodology because they thought it could provide a “*high standard of proof*” that would reassure the funders.

Contacts members of the squat had with actors in the Welfare Ministry could also be considered a guarantee of sorts:

“*When we do research, experimentation, I advocate randomisation. Either you do research or you don’t. For me, randomisation was important. The more off-the-wall the hypothesis, the more you need scientific rigor. There is no more neutral approach to programme evaluation than randomisation* […]. *Politicians and administrators want numbers. For them, qualitative data is just verbiage*.”

Within the cabinet of the Health Minister, the experimental and scientific nature of the project was, in retrospect, perceived as a major source of legitimacy, as one of her advisors explained:

“*Everything about the project was new. It was designed as an experiment, which was quite new for France, and that was good. What I liked was the really scientific aspect, with international studies, statistics. There was a whole research team behind them, it was well thought out, with lots of statistical tools*.”

In fact, the Minister, who had a scientific background, was explicit: “*A randomised trial? It’s the only way to advance in this field. The amount of amateurism and the unreliable nature of much health research is obvious.*”

### The agency of minor actors

The funding basis of the intervention was established after several months of discussion. A public health research laboratory in Marseille, already convinced of the scientific value of an RCT, was mandated responsibility for the evaluation, while that for steering the project was given to a new, inter-ministerial body, with the intent of breaking down barriers in public policy aimed at solving housing problems.

The decision to experiment with another public action model resulted from the coalition of certain minor players in the field of intervention, who shared common values and deployed a successful strategy for leveraging funds. These actors shared the belief that policies to counter exclusion had failed. They shared a culture of harm reduction and experimentation, a public health culture, and a culture that values user participation. In *Un Chez Soi d’Abord*, they saw an opportunity to act upon the social determinants of health, recognise the value of experience-based knowledge and continue supporting the harm reduction movement. Key figures in the central government administrative services who shared these convictions opened the way to experimentation through a ‘top-down’ study that valued a randomised, scientific design with a national scope, because they grasped the issues at stake in terms of transforming public policy. Specifically, they referred to the rise of the epidemiological paradigm in public action, beginning in the 1990s with the AIDS epidemic in France. They had contributed to this model, for which scientific evaluation had provided a privileged means of ex ante evaluation of legislative proposals for social policy [31].

In addition, the coalition deployed a strategy that favoured the possibility of reducing health costs for the state by reducing the length of hospital stays, and this in a context where budgetary choices were being increasingly rationalised.

### Agency, structures of political opportunity and the transfer of knowledge

The *Un Chez Soi d’Abord* programme took the specific shape of a randomised trial constituting the first step towards an evidence-based policy. As we have seen, the introduction in the health and social field of an RCT that was national in scope resulted from a process of translation, itself the result of an alliance between proponents of a social movement and minor actors in national government services. During the different stages of the experimental squat and the genesis of the *Un Chez Soi d’Abord* project, however, certain protagonists’ capacity for agency seems to have been more effective than structures of political opportunity [14]. On the one hand, two windows of opportunity allowed actors to further their cause. Municipal elections in Marseille provided a propitious moment to seek legalisation of the experimental squat, while nationally, the presidential campaign similarly enabled government supporters and others to push for *Un Chez Soi d’Abord*. On the other hand, the political environment in which the social movement against homelessness found itself is insufficient for grasping either the successful transformation of an initially local experiment into a national policy of experimentation or the choice of a RCT as the experiment to undertake at the national level.

This process, however, also led to a transformation in the status of the cause being defended. It is striking to note the manner in which players were able to form a coalition both locally and within government administrations and to exploit a representation of their cause as a viable technical solution validated by scientific expertise (i.e. being housed improves health and access to care), when other players would have gladly given precedence to the political argument (i.e. everyone has a right to housing and to healthcare). The cause originally defended through the experimental squat was so transformed, or ‘lost’, in translation as to be unrecognisable to those who had been present on the ground since the squat’s origins. As actors who advocated horizontal decision-making, many could no longer relate to the inter-ministerial project, vertically steered by the state and financed primarily with health funds. Neither could they recognise their ethical position in the RCT evaluation, which randomly assigned participants in the *Un Chez Soi d’Abord* study to experimental (i.e. housing first, with no treatment criteria) and control groups (i.e. housing and treatment ‘as usual’). While local activists had been present when the various parties were brought together, some reluctantly gave in to the randomisation scheme while others flatly rejected it:

“*I disapprove of the idea of random selection. If you interview me, you will hear me yell and scream. It is absolutely stupid to say, ‘we’re going test a control group and another group and we’re going to see who comes out best’. So you cut off a rat’s paws and compare it with a rat that has paws. You don’t need to do an experiment to figure out who will come out best!*”

“*Say I have a patient. He’s going to die in the streets, from AIDS, and he is in the control group. That really gets to me! … Do you really need a control group to show that when someone doesn’t have housing …?*”

To be persuasive, *Un Chez Soi d’Abord* proponents chose to promote the evaluation through scientific expertise of its potential economic viability rather than the right to housing or the use of knowledge based on the experience of persons with histories of homelessness and psychiatric disorders. The transformation of the original experiment, a therapeutic squat, from a participatory object into a scientific object succeeded in terms of the decision to experiment with a new way of supporting homeless persons with severe psychiatric disorders, but it failed in relation to the principle of participation. This happened for several reasons, including that the time-frame of the political agenda did not permit the elaboration of a participatory type of study, and that the minority coalition decided to focus on the scientific argument that seemed the most effective for persuading the wariest administration and at securing funding corresponding to the nature of the experiment (‘scientific’ funds). Finally, scientific backing was also a strategy for shifting the balance of power away from the ministries by mandating the RCT leadership to a new inter-ministerial actor, the *Délégation Interministérielle à l’Hébergement et à l’Accès au Logement* (Interministerial Delegation for Access to Shelter and Housing), which assures the coordination of national services for access to housing for the homeless. The latter sought institutional and political legitimacy from the IRESP, which was charged with scientific responsibility for the project.

## Discussion

To understand how the experience of a local, grass root therapeutic squat could influence the establishment of a scientifically based ‘gold standard’ experiment of national scope, the actors, processes and context had to be examined simultaneously. This paper has identified a multitude of actors, the different grass roots, administrative and professional cultures that shaped their viewpoints, and how many were nevertheless able to join together in a single coalition to bring about the RCT.

The back-story of this process highlights three historical moments that provide the context. The first was the social movement to end homelessness, beginning in 2006, which eventually brought together homeless people with NGOs, professionals and government decision-makers. The experimental squat was founded in Marseille at this time. Two other important time-points provided ‘windows of opportunity’ for persuading decision-makers in power to support the causes of activist – one was the municipal election in Marseille, during which city public health leaders joined in negotiations and the Mayor approved the legalisation of the squat, and the other was the 2012 presidential election campaign, which contributed to mobilising health ministries, whose heads and cabinets are appointed, to supporting a Housing First-type programme of national scope.

Retracing the role of minor actors in this historical process has allowed us to understand the domains of social action and culture of social worlds from various viewpoints, ranging from those of local activists to those of government ministries. In either direction, actions and mindsets required translation to bring about an innovative policy change. The study results nevertheless illustrate both the possibilities for and limits to the power of minor players to affect changes in their image, at least in the context of establishing a programme as radically different for France at the time as the *Un Chez Soi d’Abord* RCT. The capacity for agency of minor players set on politicising forms of injustice that social and psychiatric institutions cannot halt lay in their ability to form a common front around a technical solution to be validated by scientific studies. As ‘mediators’, these players were able to bring about the transfer of knowledge between distant social worlds, thereby giving meaning and scope to a public problem through a series of actions not limited to monopolies of expertise and decision-making [32]. However, in the process, principles such as horizontal decision-making, adequate user participation and the ethics of equal access to resources were subordinated to the success of the RCT model.

## Conclusion

Two epistemologies influenced the experimental framework for Housing First in France. The first, historically dominant in the medical and epidemiological fields, operates under a regime of evidence based on ‘gold standards’ like the RCT. The other attempts to transform practice by understanding and valorising the experiences, or trials and tribulations, that marginalised individuals experience [33]. After 4 years of experimentation, a huge gap could be noted between the activist experiences of the Marseilles squat, where people involved participated in a real way in everyday decisions, and the way in which the randomised *Un Chez Soi d’Abord* trial came about. In the latter case, participation developed with difficulty, despite it having been the initial plan hatched through a process of user representation in the management and direction of the study on the one hand, and through a committee of users and researchers on the other.

In actual practice, it was the greater weight given to the cost-effectiveness analysis of the experiment, which had become a strategy of the decision-makers that paid off. However, it ran the risk of unintentionally expressing an authority of the scientists that was barely open to debate and hence could not be countered by the original activists. Furthermore, those who were homeless and living with psychiatric disorders occupied a subordinate position from which they were unable to construct, let alone enter, a public arena of discussion where points of view and experiences of injustice could be confronted [34]. Although user experiences have been built into the actual RCT protocol, the politics of developing the RCT programme itself cannot be considered a collaborative study, in particular because it over-valorises the dominant scientific, i.e. non-collaborative, expertise [35]. Hence, the successful transformation of a local social movement experiment into a public policy nevertheless failed to assure the crucial participation principle of those most concerned by the construction of social responses. This raises the question of whether scientific tools are methodologically capable of integrating collaborative studies with marginalised people.

## Footnotes

1 We accept Lascoumes’s critique that cognitive frameworks ‘travel’ with far more difficulty than objects in the scientific field studied by Actor-Network-Theory. However, for ease of use we prefer the Actor-Network-Theory term ‘translation’ to Lascoume’s ‘transcoding’ [33].
